# Hypertension-related, calcium-regulated gene (HCaRG/COMMD5) and kidney diseases: HCaRG accelerates tubular repair

**DOI:** 10.1007/s40620-014-0054-3

**Published:** 2014-02-11

**Authors:** Hiroyuki Matsuda, Pavel Hamet, Johanne Tremblay

**Affiliations:** Centre de recherche, Centre hospitalier de l’Université de Montréal (CRCHUM), Montreal, QC Canada

**Keywords:** Acute kidney injury, Copper metabolism MURR1 domain-containing proteins, Hypertension-related calcium-regulated gene, p21, Re-differentiation, Renal repair

## Abstract

Hypertension is a risk factor for renal impairment. While treatment of hypertension provides significant renal protection, there is still an unmet need requiring further exploration of additional pathogenetic mechanisms. We have found that the hypertension-related, calcium-regulated gene (HCaRG/COMMD5) is involved in renal repair. HCaRG is a small intracellular protein of 225 amino acids and its gene expression is negatively regulated by extracellular calcium concentrations. HCaRG is mostly expressed in the kidneys, with higher levels found in the spontaneously hypertensive rat than in normotensive rats. In an acute kidney injury model, HCaRG expression decreases immediately after injury but increases above baseline during the repair phase. In cell cultures, HCaRG has been shown to facilitate differentiation and to inhibit cell proliferation via p21 transactivation through the p53-independent signaling pathway. Induction of p21 independently of p53 is also observed in transgenic mice overexpressing HCaRG during the repair phase after ischemia/reperfusion injury, resulting in their improved renal function and survival with rapid re-differentiation of proximal tubular epithelial cells. In addition, transgenic mice recover rapidly from the inflammatory burst most likely as a result of maintenance of the tubular epithelial barrier. Recent studies indicate that facilitating re-differentiation and cell cycle regulation in injured renal proximal tubules might be important functions of HCaRG. We have proposed that HCaRG is a component of differential genetic susceptibility to renal impairment in response to hypertension.

## Introduction

High blood pressure is a risk factor for renal failure. It is well known that malignant hypertension leads to kidney injury and that mild to moderate chronic hypertension can accelerate the progression of renal disease. Paradoxically, the most used animal model of human hypertension, the spontaneously hypertensive rat (SHR), is relatively resistant to renal damage not only when compared to other hypertensive rat models, such as the Dahl salt-sensitive rat or the Munich hypertensive strain, but also in comparison to normotensive rats such as the Brown–Norway (BN) rat, suggesting that genetic factors may affect the susceptibility to hypertension-induced or -accelerated renal disease. This has been well demonstrated by elegant studies of Theodore W. Kurtz’s group when they transplanted a kidney of the BN rat to the uninephrectomized SHR: it resulted in more severe kidney damage to the BN donor kidney than to the SHR kidney [1]. Our review focuses here on a hypertension-related gene, hypertension-related, calcium-regulated gene (HCaRG/COMMD5) that is more expressed in kidneys of SHR than of normotensive rats, and could explain in part the resistance of the SHR kidney by its accelerating effects on renal repair.

## Discovery of HCaRG

HCaRG was initially discovered by our group in the parathyroid glands of the SHR and shown to be negatively regulated by extracellular calcium concentrations. It encodes a small intracellular protein of 225 amino-acids [2]. Incubation of parathyroid cells under the low extracellular calcium condition increases HCaRG expression in the same manner as parathyroid hormone. Other hormones, such as natriuretic peptides and vasopressin, have also been shown to respond to low extracellular calcium concentrations, but HCaRG is the first intracellular protein reported to have this property, and all these genes, including HCaRG, possess a negative calcium-responsive element in their promoters [3].

Hypertension is one of the risk factors not only for kidney injuries but also for renal cell carcinoma (RCC) [4–7]. Genetic hypertension is characterized at birth by suppression of apoptosis and increment of cell proliferation, leading to neonatal heart and kidney hyperplasia [8]. During the period of hypertension development, apoptosis is increased in response to the increment of blood pressure but so is proliferation, resulting in acceleration of cell turnover. The HCaRG gene exists in one copy on human chromosome 8q24.3 with paralogs on syntenic regions of rat chromosome 7 and mouse chromosome 15. This locus is associated with kidney weight (KW), urinary calcium excretion in rats and oncogenesis in humans [9, 10]. Chromosome 8 abnormalities, which might be of particular pathogenic importance, can be detected in 15 % of patients with acute myeloid leukemia [11, 12]. Gains and high-level gene amplification at 8q have been reported in renal cell carcinoma (RCC), metastatic prostate cancers, sporadic colorectal carcinomas and metastatic gastrointestinal stromal tumors [13–18]. Chromosome 8 anomalies in certain cancers could disrupt the HCaRG gene. In fact, HCaRG levels are decreased in various cancer cell lines [2]. HCaRG levels are more abundant in the kidneys, heart and adrenal glands. HCaRG is mainly located in renal proximal tubules (RPTs) of kidneys. We became interested in the possible roles played by HCaRG in the process of kidney development and renal repair after injury.

## HCaRG as a member of the COMMD protein family

HCaRG has been assigned to a novel copper metabolism MURR1 domain-containing (COMMD) protein family, based on the identification of a well-conserved and unique domain at its carboxy-terminal end the COMM domain. The COMMD protein family has 10 members, and HCaRG corresponds to COMMD5. The length of the COMM domain varies between 70 to 85 amino acids across members of the family. The prototype of the COMMD protein family, COMMD1, exerts the function of blocking nuclear factor of kappa light polypeptide gene enhancer in B-cells (NF-κB)-chromatin interaction which, in turn, is induced by various stimuli, including tumor necrosis factor (TNF) and interleukin (IL)-1β [19, 20]. It has been determined that all members of the COMMD family are capable of inhibiting NF-κB transcriptional activity to various degrees and that the COMM domain is critical in the process. Recently, we reported that transgenic (Tg) mice overexpressing HCaRG/COMMD5 in RPTs recovered rapidly from the inflammatory burst that increased TNF-α and IL-1β as well as infiltration of macrophages after acute kidney injury (AKI) [21].

**Fig. 1:**
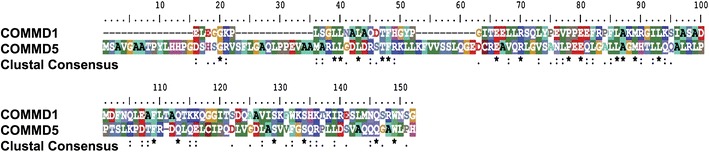
Alignment of human HCaRG/COMMD5 and COMMD1 proteins, excluding the COMM domain. While COMM domains of the COMMD protein family are highly conserved, HCaRG has only 13 % homology with COMMD1. *Asterisks* indicate positions with a single, fully-conserved residue. *Colons* depict strongly similar properties—scoring >0.5 in the Gonnet PAM 250 matrix. *Periods* indicate weak similarity—scoring ≤0.5 in the Gonnet PAM 250 matrix. *HCaRG* hypertension-related calcium-regulated gene, *COMM* copper metabolism MURR1

The COMM domain, which defines all COMMD proteins and is well conserved among members of this protein family, provides a critical interface for protein–protein interactions. It has been established that COMMD proteins are highly interactive and that the COMM domain mediates COMMD1-COMMD1 homodimerization as well as binding to other COMMD proteins [22]. While the COMM domains of COMMD1 and HCaRG are highly conserved, 87 % of amino acids differ between HCaRG and COMMD1 outside this domain, suggesting that members of the COMMD protein family could have different functions (Fig. 1).

## HCaRG as cell cycle regulator

Renal proximal tubules repair after injury requires de-differentiation, proliferation and migration of surviving tubular cells to replace dead cells [23, 24]. The process of tubular repair is similar to renal development in the fetal stage and includes a high cell proliferation rate, soaring apoptosis and a specific gene expression pattern. Interestingly, Joseph V. Bonventre’s group has demonstrated that epithelial cell cycle arrest at G_2_/M during the injury phase results in the activation of c-jun NH_2_-terminal kinase signaling, which acts to up-regulate the production of fibrogenic cytokines, thus accelerating progression of chronic kidney disease (CKD) [25]. In contrast, inhibition of cell proliferation with cycle arrest at G1 mediated by rosiglitazone, which has been used to decrease insulin resistance by activating peroxisomal proliferator-activated receptor, facilitates the recovery of proximal tubular epithelial cells (PTECs) after cisplatin-induced injury through the down-regulation of extracellular signal-regulated kinase and Akt signaling [26].

**Fig. 2:**
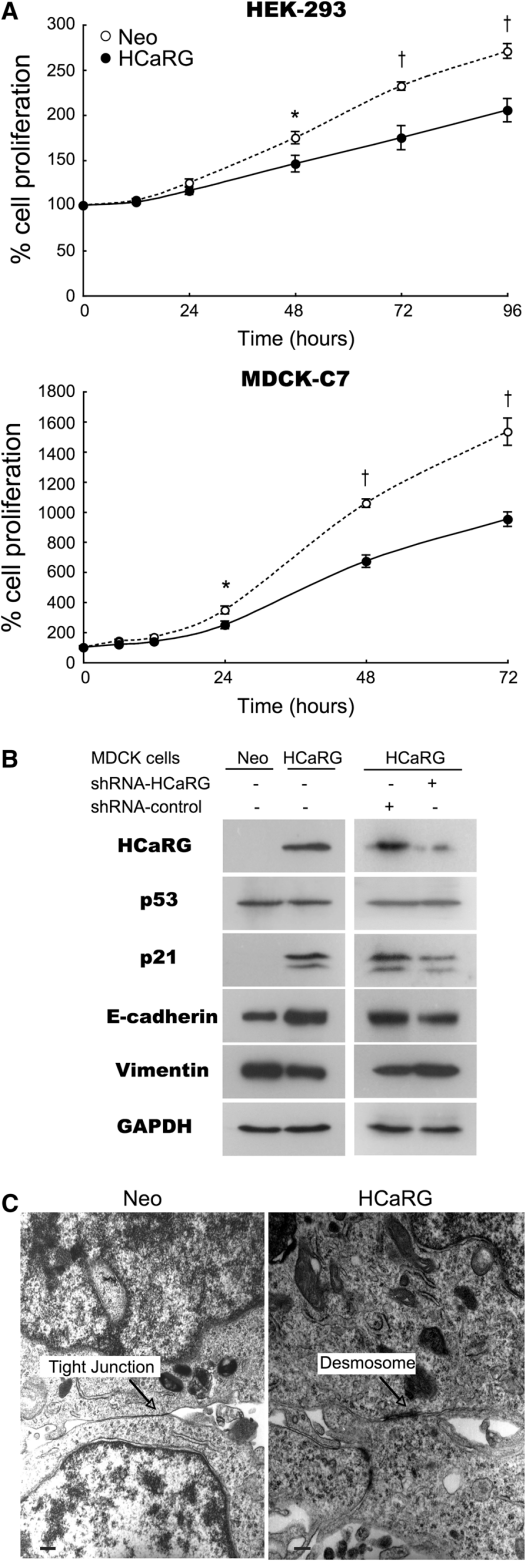
HCaRG controls cell proliferation and facilitates cellular differentiation. **a** Cell proliferation in Neo- or HCaRG-plasmid-transfected HEK-293 and MDCK-C7 cells. HCaRG inhibited cell proliferation in both kidney cell lines *p < 0.05, ^†^p < 0.01. **b** Protein expression of HCaRG, p53, p21 and differentiation phenotype markers (E-cadherin and vimentin) in Neo- and HCaRG-plasmid-transfected MDCK-C7 cells with or without shRNA. HCaRG was detected only in HCaRG-MDCK cells. HCaRG-MDCK cells showed more mature gene expression patterns than Neo-MDCK cells. HCaRG induced p21 transactivation without p53 induction. shRNA-HCaRG treatment lowered HCaRG levels compared to a non-target control. HCaRG reduction led to lower p21 expression and abolition of differentiation in HCaRG-MDCK cells. Data from Matsuda et al. [21]. **c** Electron microscopy of Neo- and HCaRG-plasmid-transfected HEK-293 cells. HCaRG-HEK-293 cells had mature desmosome-like junctions instead of the tight junctions of Neo-control cells. *Scale bars* 300 nm. Figure from Devlin et al. [28]

The effect of HCaRG on cell proliferation was demonstrated in two kidney cell lines, human embryonic kidney (HEK)-293 cells and Madin–Darby canine kidney (MDCK)-C7 cells [27]. The cells were stably transfected with either control plasmid (Neo) or HCaRG expression plasmid (HCaRG). HCaRG-overexpressing cells presented a much lower proliferation rate (Fig. 2a) and DNA synthesis than control cells. In addition, HCaRG delayed the cell cycle with accumulation of cells at G_2_/M, without arrest [28].

The gene expression profiles of 111 cell cycle regulatory genes in stably-transfected cells have been analyzed [28]. HCaRG overexpression markedly induced p21 and down-regulated p27 in HEK-293 cells. HCaRG overexpression also augmented p21 expression independently of p53 expression in MDCK-C7 cells (Fig. 2b) [21]. This up-regulation was diminished by HCaRG knockdown with short-hairpin RNA (shRNA). p21, accompanied by p53 up-regulation, has been demonstrated to be induced in cells undergoing either p53-dependent G_1_ growth arrest or apoptosis [29]. p21 levels can also rise rapidly by differentiation inducers through a p53-independent pathway. In that case, p21 induction is coupled with the expression of early differentiation markers [30]. p21, independently of p53, could act as an inducible growth inhibitor that contributes to slow down the cell cycle and facilitates differentiation [31].

## HCaRG fosters cellular maturation

HCaRG has been associated with changes in cell morphology and the appearance of differentiated phenotypes, namely, HCaRG-overexpressing HEK-293 cells present features of mature epithelial cells, such as higher protein content, cell size and volume [28]. Transmission electron microscopy ultrastructural analysis has revealed the presence of more differentiated junctions (desmosome-like) in HCaRG-overexpressing cells than in Neo controls, and these cells demonstrate features consistent with junctional (glandular-like) complex formation and numerous microvilli (Fig. 2c). These cellular changes are concordant with several modifications in the levels of specific epithelial/mesenchymal markers. E-cadherin, a marker of epithelial integrity [32], is higher in HCaRG-overexpressing cells than in control cells (Fig. 2b) [21]. In contrast, vimentin, an intermediate filament protein that is only expressed in mesenchymal cells [33], is lower in HCaRG-overexpressing cells. These observations indicate that HCaRG could accelerate differentiation and maturation via p21 transactivation through a p53-independent pathway in kidney cells, resulting in delay of the cell cycle with G_2_/M accumulation.

## HCaRG stimulates renal cell migration

Acute kidney injury evokes actin cytoskeleton disruption and aggregation by induction of cofilin and, consequently, causes the breakdown of PTEC apical membrane microvilli during the early stage after ischemia [34]. Aquaporin-1, which is widely expressed in epithelial and endothelial cell membranes and facilitates transepithelial water transport, accelerates the migration of PTECs by actin re-organization, resulting in decreased tubular injury after ischemia [35]. Such cytoskeletal organization and migration of PTECs are key events occurring during tubular injury and repair.

**Fig. 3:**
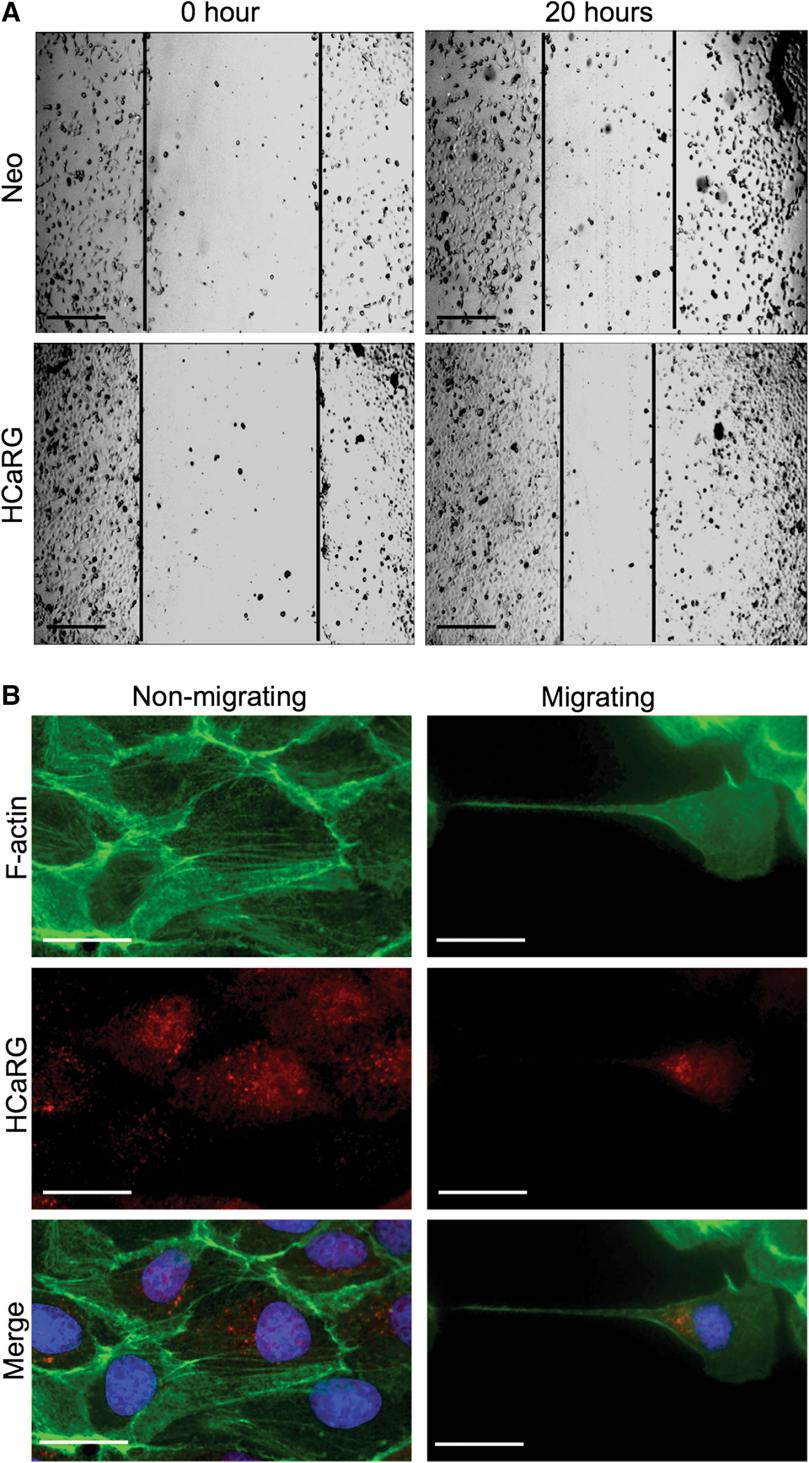
HCaRG stimulates renal cell migration. **a** Wound healing assay in Neo- or HCaRG-plasmid-transfected HEK-293 cells. HCaRG markedly accelerated wound healing after 20 h compared to Neo-controls. *Scale bars* 50 μm. **b** Intracellular location of F-actin (*green*) and HCaRG (*red*) in migrating MDCK cells during the wound-healing process. In non-migrating cells, HCaRG is located in the perinuclear space, and organized actin fibers are clearly visible. In the migrating cell, intracellular actin fibers are de-polymerized and short F-actin filaments are observed in the perinuclear space. HCaRG localizes with short F-actin filaments between the nucleus and elongated tip. Nuclei are stained by DAPI (*blue*). *Scale bars* 25 μm (color figure online)

As described previously, HCaRG accelerates differentiation and maturation via p21 transactivation. On the other hand, HCaRG overexpression increases the motility of kidney cells through the secretion of transforming growth factor-α in the wound-healing assay (Fig. 3a) [36]. We recently examined the intracellular location of HCaRG and actin in migrating MDCK-C7 cells during wound healing (Fig. 3b). In non-migrating cells, we observed HCaRG localization in the perinuclear space and clearly visible actin fibers with no signs of interactions between the two proteins. In migrating cells, intracellular actin fibers disappeared, and short filamentous (F)-actin accumulation was seen between the nucleus and elongated tip. HCaRG was also seen concentrated with short F-actin between the nucleus and elongated tip. It is known that in migrating fibroblasts, centrosomes reorient between the nucleus and elongated tip, which in turn re-positions the Golgi apparatus that is thought to establish and maintain cell polarity during migration [37–40]. We postulate that HCaRG could play a role in stimulating cell migration and wound healing without de-differentiation, by directly involving actin re-organization.

## HCaRG accelerates renal tubular repair after injury

### Acute kidney injury

Ischemia/reperfusion injury (IRI) causes acute tubular damage, one of the most common forms of human AKI. After renal ischemia with renal hypoperfusion and hypoxia, PTECs lose their structural integrity through a sequence of events that include the disruption of brush border with blebbing of the apical membrane, fragmentation and internalization, and a rapid change in cell polarity. During the recovery phase, surviving PTECs proliferate and replace the irreversibly injured PTECs, migrate to cover the exposed areas of the basement membrane and re-differentiate to restore tubular integrity [23]. In a rat IRI model, we determined that HCaRG mRNA levels decreased soon after reperfusion and reached their lowest levels after 3–6 h, when PTECs were de-differentiated and proliferated. HCaRG mRNA levels then rose steadily to higher than baseline 48 h after reperfusion, corresponding to the regeneration phase [2].

Tg mice overexpressing HCaRG in RPTs were generated to test the hypothesis that HCaRG could stimulate renal repair [21]. Exogenous HCaRG was inserted into the kidney androgen-regulated protein (KAP) promoter plasmid. KAP was identified as an abundant protein under androgen control and expressed in RPTs [41, 42]; therefore, exogenous HCaRG was expressed in RPTs of Tg male mice under androgen control.

In an AKI model, IRI caused severe tubular damage that resulted in pronounced renal dysfunction in both non-Tg and Tg mice [21]. The survival rate 7 days after IRI injury was 64 % in Tg compared to only 25 % in non-Tg mice (p = 0.0249). In addition, morphological tubular damage and renal function in Tg mice improved significantly faster in comparison to non-Tg mice after 2 days (Table 1). We concluded that HCaRG overexpression significantly enhances renal function and survival after ischemia.

**Table 1:** Renal function 2 days after ischemia/reperfusion injury (IRI)

Genotype	Blood urea nitrogen (mmol/l)	Creatinine (μmol/l)
Non-Tg mice	105.0 ± 25.7 (n = 10)	206.1 ± 99.1 (n = 10)
Tg mice	77.4 ± 33.5 (n = 10)*	114.4 ± 63.6 (n = 10)*

Data are shown as mean ± SD. *p < 0.05

**Fig. 4:**
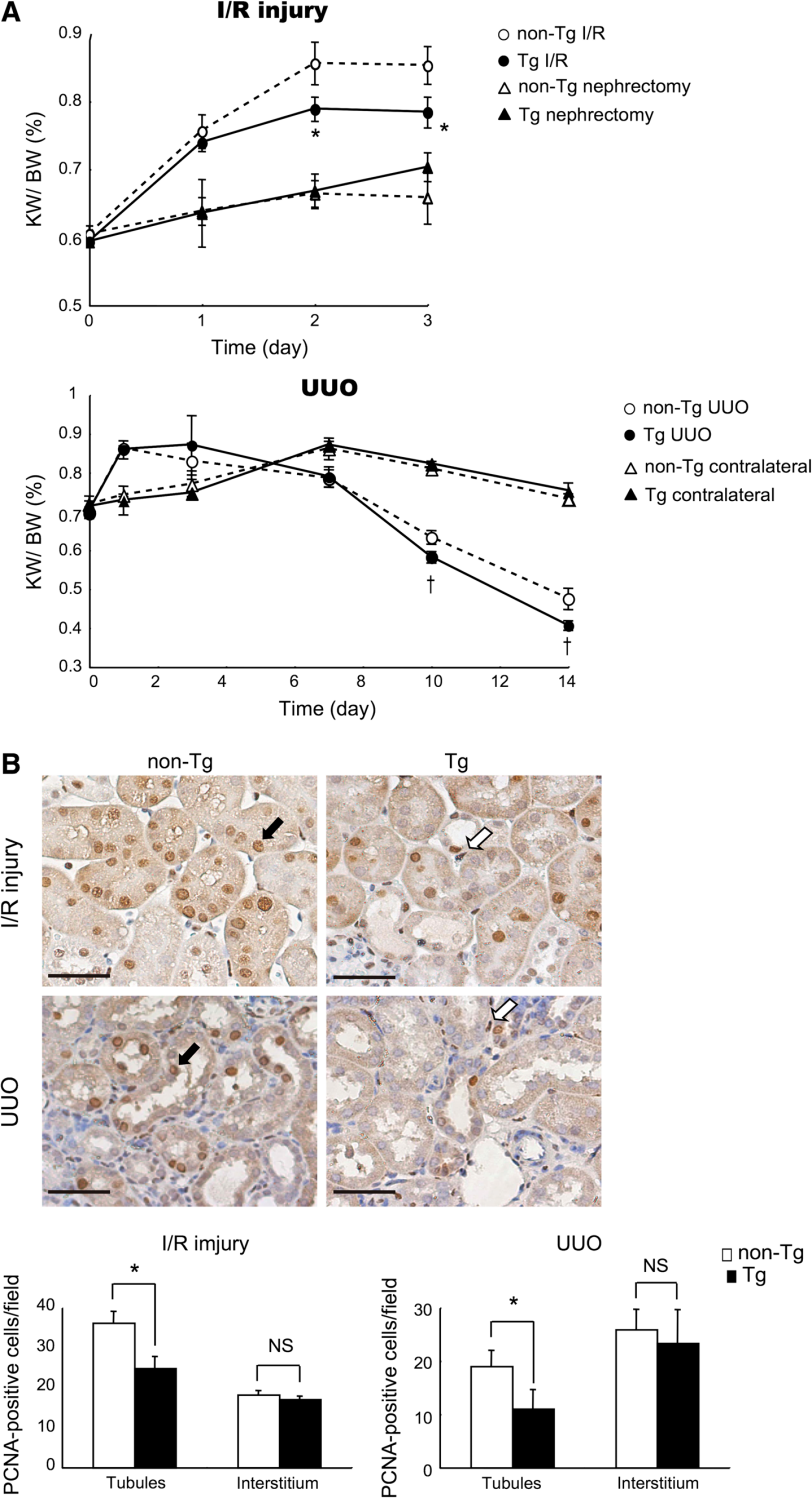
HCaRG-Tg mice exhibit lower tubular cell proliferation after ischemia/reperfusion injury (IRI). **a** Changes in the kidney weight (KW)/body weight (BW) ratio after IRI and unilateral ureteral obstruction (UUO). IRI increased KW compared to nephrectomized controls, and this increment was significantly reduced (*p < 0.05) in Tg mice after 2 days. In the UUO model, KW increased with hydronephrosis caused by ureteral obstruction for up to 3 days. After day 7, KW started to decline, and HCaRG elicited a significantly more rapid reduction of KW (^†^p < 0.05). **b** Localization of proliferating cell nuclear antigen (PCNA)-positive cells on day 2 after IRI and on day 10 after UUO. The number of PCNA-positive cells was counted in tubular and interstitial regions, respectively. PCNA-positive cells in tubules were lower in Tg than in non-Tg mice. The *black arrow* indicates PCNA-positive tubular cells, and the *white arrow* depicts PCNA-positive interstitial cells. *p < 0.05. *Scale bars* 50 μm. Data from Matsuda et al. [21]

Tubular epithelial cells and cells within the interstitial space, which are associated with vascular network components and resident fibroblasts, account for about 80 % of kidney volume, and increased KW could be due to edema, hypertrophy and cell proliferation after tubular injury [43]. HCaRG overexpression did not affect renal hypertrophy by uninephrectomy, but significantly reduced the increase of KW 2 days after reperfusion (Fig. 4a). Such KW reduction could have resulted from stimulation of cell death or inhibition of cell growth. However, we demonstrated that HCaRG had no effect on apoptotic cell death after IRI. A large number of proliferating cell nuclear antigen (PCNA)-positive cells were present in tubules on day 2 (Fig. 4b). HCaRG overexpression decreased only the number of proliferating tubular cells, while the number of proliferating cells in the interstitium was comparable between non-Tg and Tg mice. This inhibition of PTEC proliferation was accompanied by p53-independent p21 transactivation. It is known that p21 transactivation by a p53-independent pathway occurs in RPTs but not in the glomerulus or interstitium during the renal recovery phase, and might thus have contributed to cell differentiation [44–46]. In p21 knockout mice, AKI causes more rapid onset of renal dysfunction, and induces more severe morphological damage with a threefold higher mortality rate than in normal mice [45, 47]. Miyaji et al. [46] reported that cisplatin-induced AKI evoked 2 peaks of increased p21. The first peak was accompanied by up-regulation of p53 and PCNA, possibly reflecting G_1_ arrest and DNA repair. The second p21 peak occurred through a p53-independent pathway likely contributing to cell differentiation. These studies indicate that p53-independent p21 up-regulation could be crucial in controlling epithelial proliferation and morphogenesis in RPTs. Actually, in HCaRG Tg mice, E-cadherin levels recovered more rapidly, while vimentin induction after IRI disappeared faster than in non-Tg mice.

### Chronic kidney disease

Tubulointerstitial fibrosis is recognized to be a common endpoint of human CKD caused by hypertension, diabetes, and nephrotic syndrome. Unilateral ureteral obstruction (UUO) is used as an animal model of progressive tubulointerstitial fibrosis. UUO also leads to many pathophysiological events of obstructive nephropathy, such as cellular infiltration and proliferation, tubular de-differentiation, apoptosis and atrophy, fibroblasts differentiation and excessive extracellular matrix deposition [43, 48].

KW is increased 3 days after obstruction because UUO produces tubular dilatation and interstitial edema (Fig. 4a) [21]. HCaRG overexpression does not reduce the initial edema in Tg mice. Contralateral KW gain until 7 days after obstruction is mainly due to hypertrophy, and HCaRG overexpression does not affect this hypertrophy, as in the uninephrectomized kidneys. Ten days after surgery, UUO results in gradual KW reduction, and HCaRG overexpression causes more rapid KW diminution in Tg mice compared to non-Tg mice. This KW reduction also derives from the imbalance of cell growth and loss. In the obstructed kidneys after 10 days of UUO, HCaRG overexpression decreases only the number of proliferating tubular cells, as after IRI (Fig. 4b).

The results in both IRI and UUO models can be summarized as follows: (1) HCaRG overexpression does not reduce initial edema, hypertrophy and injury; (2) HCaRG overexpression leads to faster KW diminution in Tg than in non-Tg mice; (3) these KW decreases are due to inhibition of cell proliferation increment in tubules but not in the interstitium; (4) HCaRG elicits re-differentiation via p21 induction after injury. In conclusion, HCaRG does not reduce initial tubular injury, but accelerates repair of RPTs which improves survival by facilitating re-differentiation of resident PTECs and controlling proliferation via p21 induction through its p53-independent pathway.

Finally, slow-cycling renal progenitor-like cells provide regenerating cells that replace injured cells during the repair phase after AKI [49, 50]. At an early phase of tubular regeneration, their daughter cells after multiple cell division show mesenchymal phenotypes such as active proliferation and migration in the interstitium, and eventually differentiate into PTECs. HCaRG could play a role in the differentiation and survival of resident renal progenitor-like cells.

## Conclusion

**Fig. 5:**
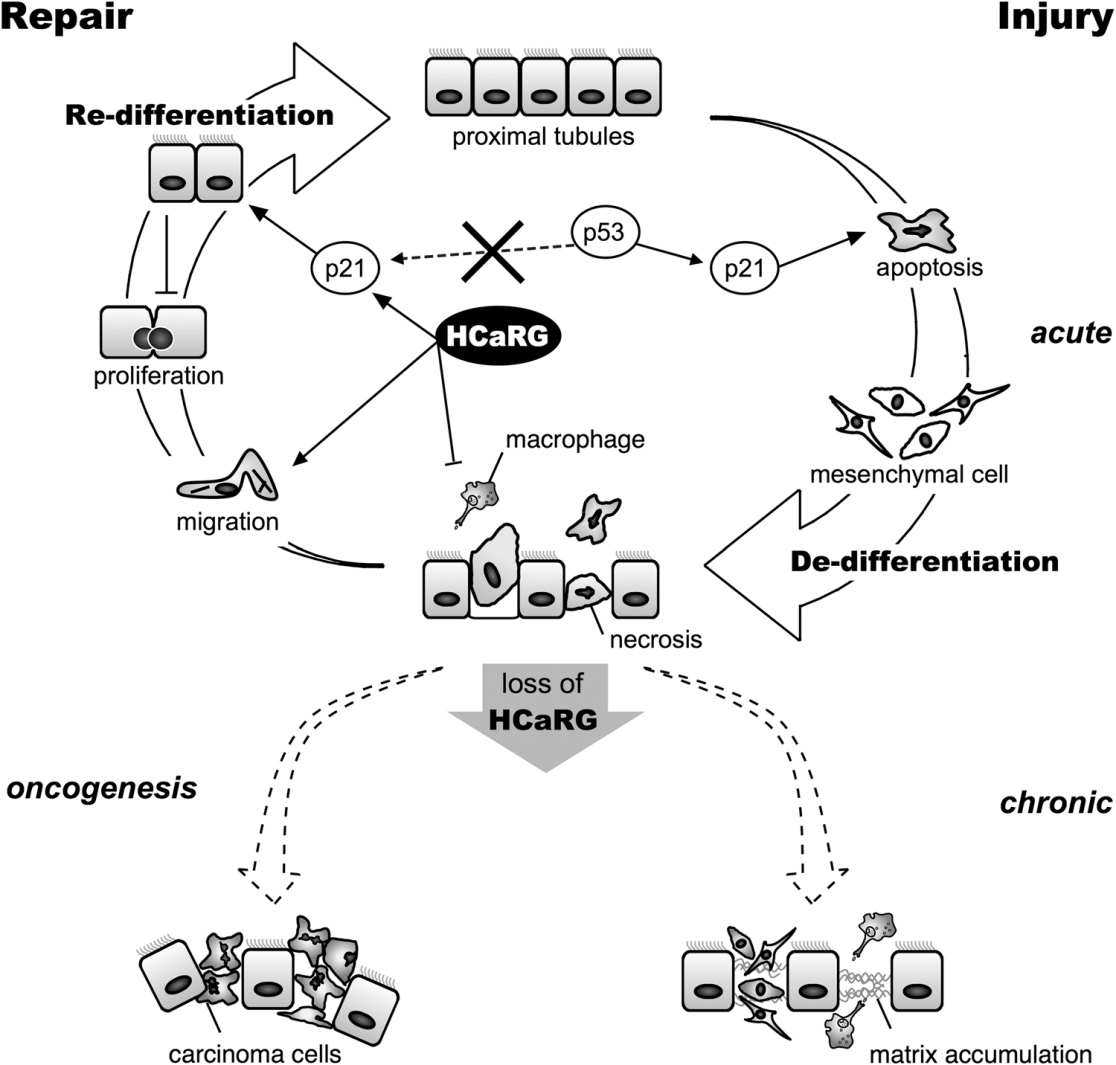
Scheme depicting the putative role of HCaRG in tubular repair after acute kidney injury (AKI). AKI mainly damages proximal tubular epithelial cells and causes cell death, resulting in necrosis and apoptosis. The induction of cell death is associated with p21 up-regulation dependent on the p53 pathway. Sublethally-injured proximal tubular epithelial cells (PTECs) de-differentiate into mesenchymal cells. De-differentiated cells proliferate and migrate to repair denuded areas. In the repair phase, HCaRG promotes cell migration, and controls PTEC proliferation by acceleration of their re-differentiation via p21 transactivation through the p53-independent pathway, resulting in improved renal function and survival. Lack of HCaRG might lead to uncontrolled cell proliferation and migration of PTECs with de-differentiated phenotypes, resulting in increased risk of chronic kidney diseases and oncogenesis of renal cell carcinoma

Acute kidney injury occurs in various clinical settings, including renal ischemia arising from septic shock and major cardiovascular surgery, or acute drug or toxic exposure. It is a common clinical problem with increasing incidence and mortality, poor prognosis, and unsatisfactory therapeutic options [51–54]. Despite better knowledge acquired during the last decade of the pathophysiological pathways underlying kidney diseases at both the basic research and clinical levels, the progression of renal dysfunction and the number of hemodialyzed patients is still increasing steadily every year. Repair after AKI involves the proliferation of PTECs as well as migration and re-differentiation. HCaRG has shown beneficial effects in facilitating re-differentiation and controlling proliferation through p21 induction via the p53-independent signaling pathway during the renal repair phase (Fig. 5). HCaRG also might stimulate the migration of PTECs during the repair phase directly involving actin re-organization. Moreover, IRI rapidly activates inflammatory responses, resulting in endothelial dysfunction in the cortex [55]. HCaRG rapidly reduces inflammatory mediators and infiltration of macrophages after reperfusion [21]. Sepsis-induced AKI has a distinct pathophysiology which involves intrarenal hemodynamic changes, vascular endothelial dysfunction, infiltration of inflammatory cells in the renal parenchyma, obstruction of tubules with apoptotic and necrotic cells, and tubular epithelial mitochondrial dysfunction [56, 57]. HCaRG may have the potential to diminish these inflammatory responses and to prevent mitochondrial dysfunction through maintenance of the tubular epithelial barrier not only after IRI but also in sepsis-induced AKI. In addition, lack of HCaRG might lead to uncontrolled cell proliferation and migration, thus increasing the risk of oncogenesis of RCC. In fact, HCaRG levels are significantly decreased in tumors of brain, kidney and liver compared to normal adjacent tissues [2] and rosiglitazone suppressed the growth of gastric cancer by up-regulating HCaRG [58]. Tubular regenerating functions mediated by HCaRG are most important for improved renal function and survival after AKI. Accelerated healing by HCaRG could serve as a new therapeutic approach to AKI.
